# An innovative network based on double receptive field and Recursive Bi-directional Long Short-Term Memory

**DOI:** 10.1038/s41598-021-01520-y

**Published:** 2021-11-26

**Authors:** Pengfei Meng, Shuangcheng Jia, Qian Li

**Affiliations:** Mogo Auto Intelligence and Telematics Information Technology Co., Ltd, Beijing, China

**Keywords:** Environmental economics, Socioeconomic scenarios

## Abstract

Sequence recognition of natural scene images has always been an important research topic in the field of computer vision. CRNN has been proven to be a popular end-to-end character sequence recognition network. However, the problem of wide characters is not considered under the setting of CRNN. The CRNN is less effective in recognizing long dense small characters. Aiming at the shortcomings of CRNN, we proposed an improved CRNN network, named CRNN-RES, based on BiLSTM and multiple receptive fields. Specifically, on the one hand, the CRNN-RES uses a dual pooling core to enhance the CNN network’s ability to extract features. On the other hand, by improving the last RNN layer, the BiLSTM is changed to a shared parameter BiLSTM network using recursive residuals, which reduces the number of network parameters and improves the accuracy. In addition, we designed a structure that can flexibly configure the length of the input data sequence in the RNN layer, called the CRFC layer. Comparing the CRNN-RES network proposed in this paper with the original CRNN network, the extensive experiments show that when recognizing English characters and numbers, the parameters of CRNN-RES is 8197549, which decreased 133,752 parameters compare with CRNN. In the public dataset ICDAR 2003 (IC03), ICDAR 2013 (IC13), IIIT 5k-word (IIIT5k), and Street View Text (SVT), the CRNN-RES obtain the accuracy of 96.90%, 89.85%, 83.63%, and 82.96%, which higher than CRNN by 1.40%, 3.15%, 5.43%, and 2.16% respectively.

## Introduction

Character recognition in natural scenes is a classic and basic task. Existing methods are mainly divided into two categories, recognition based on traditional algorithms (e.g., binarization, dilation, and erosion) and neural network algorithms^1–6^. When using the traditional algorithm, it is necessary to make more design and thinking about the noise, image quality, and resolution of the picture. With the development of neural networks, image recognition technology has made many breakthroughs. CRNN^1^ has been proven to be a popular end-to-end character sequence recognition network. After analyzing the network structure and algorithm principle of CRNN in detail, we proposed a new and improved CRNN network for the shortcomings of CRNN, and we named it CRNN-RES. Specifically, first, we designed the CRFC layer that can flexibly configure the length of the input data sequence in the RNN layer, the CRFC layer solves the problem that the recognition effect of long dense small characters is very poor, or even completely unrecognizable. Secondly, we use a cyclic recursive single-layer BiLSTM in the last RNN layer, which reduces the number of parameters without causing a decrease in accuracy. Based on extensive experiments, our proposed recursive structure can improve the recognition accuracy of the network while reducing the number of parameters. Finally, to solve the problem that the CRNN network is not friendly to wide characters, we added a wide pooling core based on the original narrow pooling core. When performing pooling operations, our method uses double-pooling cores. In this setting, our proposed CRNN-RES not only significantly decreases the network parameters but also improves the accuracy of network recognition. We experiment on four public datasets ICDAR 2003 (IC03)^4^, ICDAR 2013 (IC13)^5^, IIIT 5k-word (IIIT5k)^6^, and Street View Text (SVT). Compared with the original CRNN network, the proposed CRNN-RES achieves the goal of reducing model parameters, increasing the accuracy, and accelerating recognition speed.

## Related work

In 1985, Rumelhart and Hinton proposed a back propagation (BP) neural network^7^, which made the training of neural network much easier. In 1988, LeCun proposed the Lenet5 network^8^, Lenet5 used a convolution neural network (CNN) for the first time, using convolution, pooling, and nonlinear three layers as a series. In 2010, Dan Claudiu Ciresan and Jurgen Schmidhuber invented a neural network that can be trained on Graphics Processing Unit (GPU)^9^. In 2012, Alex Krizhevsky invented the AlexNet deep neural network^10^ and won the champion of the ImageNet competition. Subsequently, more and deeper neural networks were invented. In 2014, the University of Oxford invented the Visual Geometry Group (VGG) network^11^, and used the small convolution kernel of $3*3$ in each convolution layer for the first time. In ImageNet Large Scale Visual Recognition Challenge (ILSVRC) localization and classification competition, VGG obtained first and second place respectively. In 2015, He Kaiming invented the Residual Neural Network (RESNET) deep neural network^12^, proposed and applied the concept of residual network, which solved the network degradation problem with the increase of network level, and the neural network became deeper and had better effect. After that, neural network developed rapidly in image recognition, and various networks emerged. At present, CRNN^1^ network is widely used in the field of Optical Character Recognition (OCR). The CRNN^1^ network is widely used because it uses the network architecture of CNN plus RNN (Recurrent Neural Network), and plus transpose layer, which makes it possible to recognize the sequence of images. One feature of the sequence object is that its length is variable, and CRNN solves the recognition problem of image sequences, so CRNN has this network architecture that can recognize characters of indefinite length. It can be trained end-to-end and can meet the recognition of characters with variable length, which is one of the main reasons that CRNN is widely used in the field of pattern recognition. After studying in detail the network structure and calculation principle of CRNN, we proposed a new and improved CRNN network, in which the last two layers of BiLSTM^2,3^ of the original CRNN^1^ is changed into one layer of BiLSTM with shared parameters. Tests were carried out on four public datasets. Compared with the original CRNN network, the improved model has less model parameters, smaller model size, higher accuracy and faster recognition speed.

The remainder of the paper is organized as follows: Section II reviews the related work about our task. Section III presents the proposed method. Section IV conducts the experimental evaluation on the comparison between the proposed method and other methods. Section V concludes the paper and identifies the future work.

## Method

**Figure 1:**
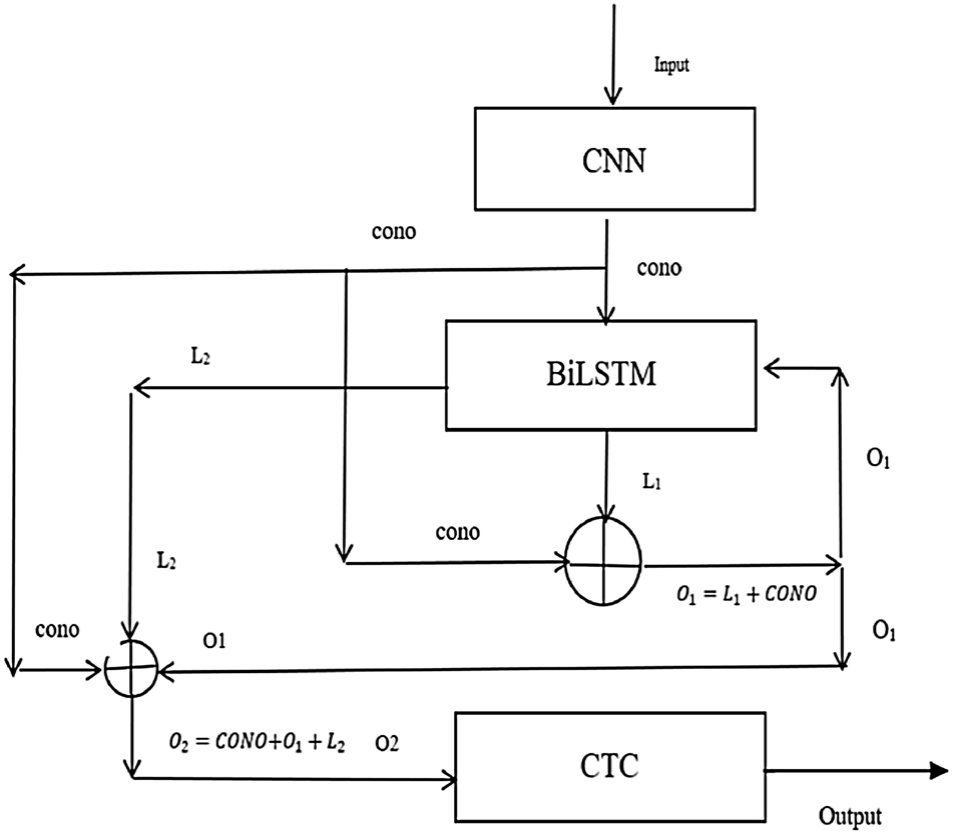
Summary architecture diagram of the RNN layer in the CRNN-RES network.

The proposed method consists of two parts: (a) The Structure of The CRNN-RES Network and The Modifications of The RNN Layer, (b) Modifications of The Convolution Layer. The structure overview is shown in Fig. 1.

### The structure of The CRNN-RES network and the modifications of the RNN layer

The overall network structure of CRNN^1^ consists of three parts: convolution layer, RNN layer, and transcription layer. From the RNN part of the CRNN-RES in Fig. 1, we can see that the RNN layer of the CRNN-RES is significantly different from the RNN layer of CRNN. In training, recursively training some parameters of the network can improve the effect of the network, and the amount of parameters is smaller than that of directly superimposing two-layer network modules. Since the original CRNN network uses two layers of BiLSTM, our goal is to reduce the overall network parameters and ensure the effect of the network. Specifically, we improve the BiLSTM of RNN layer in CRNN. We change the BiLSTM from double layer to single layer, and train the single-layer BiLSTM network block in a cyclic recursive way. The RNN part of the network structure proposed in this article is introduced as follows:

We use BiLSTM^2,3^ as the basic network of the RNN layer. BiLSTM is a two-way LSTM network, the traditional LSTM can only learn the one-way feature dependency of the image sequence, but the sequence we recognize may have a reverse dependency, such as the words “google”, “brother”, we can predict the words “google” and “brother” based on “googl” and “brothe”, or predict the words “google” and “brother” based on “oogle” and “rother”. So here we choose the bidirectional LSTM as the basic network in the RNN module of the CRNN-RES.

We use recursive training strategy, specifically, CRNN-RES adds a short-circuit connection between the output of the convolution layer and the output of the BiLSTM layer, and the result of the first output is taken as part of the input of the BiLSTM. The specific process is as follows: firstly, CONO (the output of the network in the convolution layer) is put into the BiLSTM layer, and then L1(the output result of the BiLSTM layer) and CONO (the output result of the convolution layer) are added to get a result, which is represented by O1 here. The specific process can be expressed as follows:

1 $$\begin{aligned} O_1 = L_1 + CONO \end{aligned}$$

Then we input $O_1$ into BiLSTM, and $L_2$(the result of the second BiLSTM layer) is added to $O_1$ and CONO (the output of the convolution layer), and finally, $O_2$(the final output of RNN layer) is obtained, which is expressed as follows:

2 $$\begin{aligned} O_2 = O_1 + L_2 + CONO \end{aligned}$$

The process is shown in Fig. 1. The output of the convolution layer is denoted as CONO, and the operation function of BiLSTM is denoted as F, then the first and second operations can be expressed as follows:

3 $$\begin{aligned} \left\{ \begin{array}{lr} O_1= CONO +F(CONO)\\ O_2 = F(O_1) +CONO +O_1 \end{array} \right. \end{aligned}$$

The specific process of the RNN layer is as follows: we input data which has a shape of [*timestep*, *batchSize*, 512] firstly to the BiLSTM, and we will get an output data which has a shape of [*timestep*, *batchSize*, 512]. Then we add the previous output of the BiLSTM to the output of the convolution layer, and the output shape is [*timestep*, *batchSize*, 512]. The previous output result is input to BiLSTM again, the input shape of data is [*timestep*, *batchSize*, 512], the output shape is [*timestep*, *batchSize*, 512].

The second BiLSTM operation results are then added to the previous two outputs, and then the output dimension of the data is changed by the full connection layer. The final shape of the output is $[timestep * batchSize, nclass]$, finally we convert the output, the final output shape is [*timestep*, *batchSize*, *nclass*]. Here timestep refers to the length of time series, batchSize refers to the number of each batch of pictures input to the network during training, and *nclass* refers to the number of categories of classification.

### Mathematical theory of RNN layer

**Figure 2:**
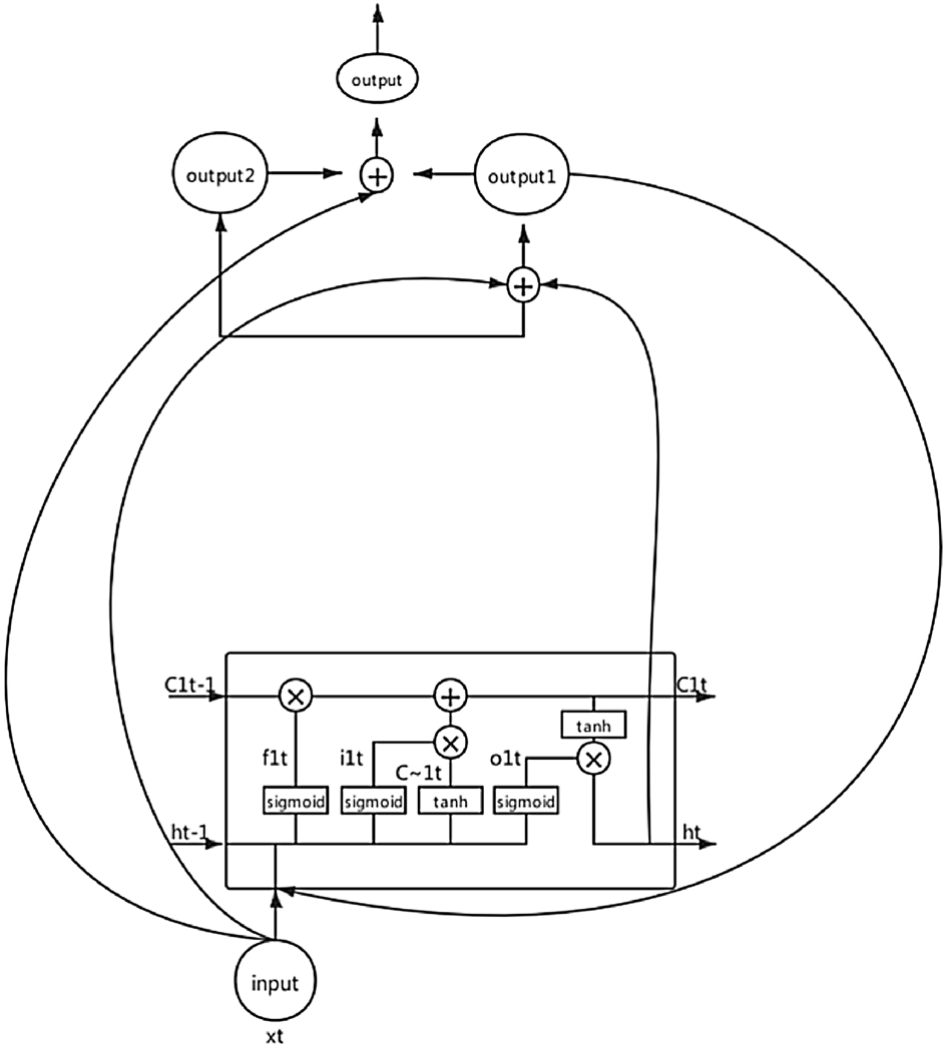
The architecture of one-way LSTM.

For CRNN-RES networks, the calculation principle is the same as LSTM^3^ or BiLSTM. In order to make the elaboration of the calculation principle more concise and convenient for readers to understand, we only calculate the form of one-way LSTM. All mathematical calculations are based on the following network architecture. As show in Fig. 2.

1) Calculate the information of the forgetting gate:

4 $$\begin{aligned} f_{1t} = sigmoid(w_f * [h_{t-1},x_t] + b_f) \end{aligned}$$

Among them, $w_f$ represents the weight, $b_f$ represents the bias term, $h_{t-1}$ is the output of the last hidden unit of lstm, $x_t$ is the input of the current hidden neural unit, and we use sigmoid as the activation function.

2) Calculate the information of the memory gate:

5 $$\begin{aligned} i_{1t} = sigmoid(w_i[h_{t-1},x_t] + b_i) \end{aligned}$$

Among them, $w_i$ represents the weight, $b_i$ represents the bias term. $h_{t-1}$ is the output of the last hidden unit of lstm, $x_t$ is the input of the current hidden neural unit, and we use sigmoid as the activation function.

6 $$\begin{aligned} C_{1t} = tanh(w_c[h_{t-1},x_t] + b_c) \end{aligned}$$

Among them, $w_c$ represents the weight, $b_c$ represents the bias term.

3) Calculate the neural unit state at the current moment:

7 $$\begin{aligned} C_{1t} = C_{1t-1} * f_{1t} + i_{1t} *C_{1t} \end{aligned}$$

Where $C_{1t-1}$ represents the state of the cell at the previous moment.

4) Calculate the information of the output gate:

8 $$\begin{aligned} O_{1t} = sigmoid(w_o[h_{t-1},x_t] + b_o) \end{aligned}$$

Among them, $w_o$ represents the weight, $b_o$ represents the bias term.

5) Calculates the current state of the current hidden layer:

9 $$\begin{aligned} H_t = O_{1t} * tanh(C_{1t}) \end{aligned}$$

Where $C_{1t}$ represents the state of the cell at the current moment. At this time, the calculation of LSTM unit is completed.

6) Next, perform the following calculations:

10 $$\begin{aligned} output1 = x_t + h_t \end{aligned}$$

Where $x_t$ is the input of the current hidden layer neural unit, $h_t$ is the output of the current hidden unit of lstm. Re-input output1 into the lstm unit. Then there are:

11 $$\begin{aligned} \left\{ \begin{array}{lr} f_{1t} = sigmoid(w_f * [h_{t-1},output1] + b_f)\\ i_{1t} = sigmoid(w_i[h_{t-1},output1] + b_i)\\ C_{1t} = tanh(w_c[h_{t-1},output1] + b_c)\\ C_{1t} = C_{1t-1} * f_{1t} + i_{1t} *C_{1t}\\ O_{1t} = sigmoid(w_o[h_{t-1},output1] + b_o)\\ H_t = O_{1t} * tanh(C_{1t})\\ output2 = x_t + h_t \end{array} \right. \end{aligned}$$

The final output as follow:

12 $$\begin{aligned} output = x_t +output2 +output1 \end{aligned}$$

### CNN and RNN flexible convergence layer

In CRNN, the general approach is to directly input the features of the CNN layer output to the RNN layer after dimensional transformation. We assume that the shape of the input data of the network is [8,1,32,128], and 8 is represented as 8 pictures for each batch, 1 is represented as 1-channel grayscale picture, 32 is represented as the width of the picture, and 128 means the hight of the picture. Then after the CNN layer of CRNN, the output feature shape is [8,512,1,16]. At this time, CRNN’s method is to convert the feature into a feature of shape [16,8,512], and then input features with sequence length of 16 into the RNN layer. In this setting, there are two problems:

(a) If the maximum character length in all pictures is 2, and each picture contains at most 2 characters that need to be recognized, then the features will also be divided into 16 sequences and sent to the RNN layer, but in fact, we only need to two sequences are sufficient for prediction. Feeding 16 sequences will undoubtedly increase the parameters of the RNN network. In practice, we do not need 16 sequences, only two sequences are enough. Too many sequences will reduce the accuracy and convergence speed of the network.

(b) If the minimum character length in all pictures is 17, and each picture contains at least 17 characters that need to be recognized, then the features will also be divided into 16 sequences and sent to the RNN layer, and the network will finally get the accuracy of each sequence. That is the maximum length of the recognition result of the current picture is only 16 characters, which means that the recognition rate of the network will always be 0 at this time, and it will never converge.

**Figure 3:**
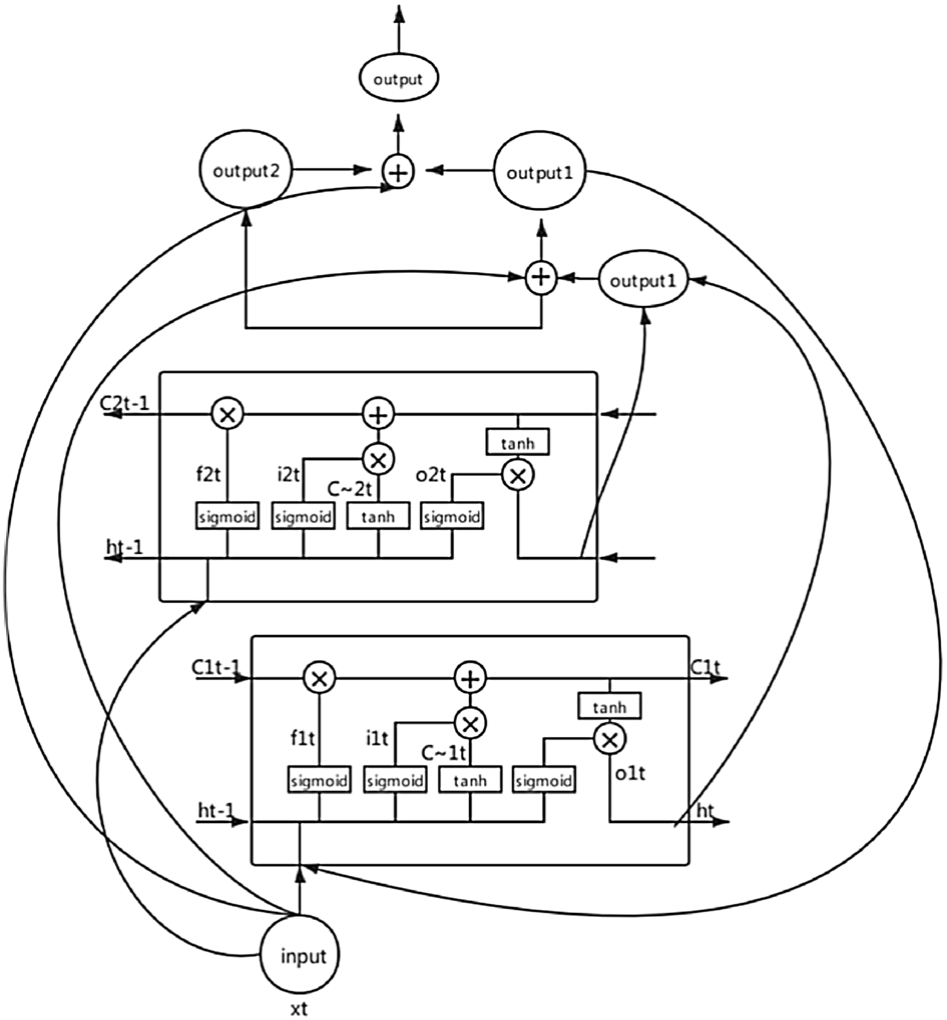
Detailed architecture diagram of the RNN layer in the CRNN-RES network.

**Figure 4:**
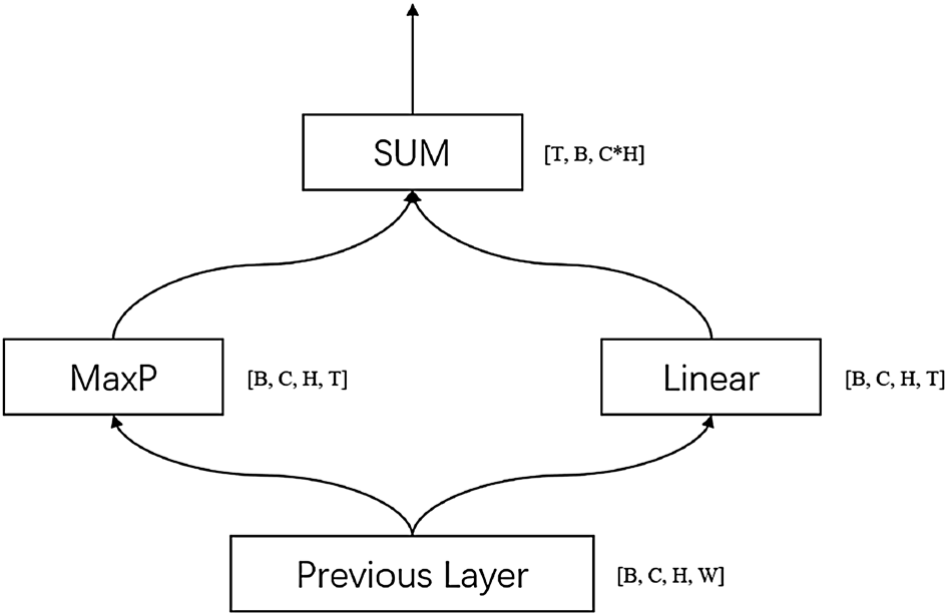
The structure of CNN and RNN flexible convergence layer. Because pooling layer is location dependent and full connection layer is location independent, in order to integrate the characteristics of location dependent and location independent, we use pooling and full connection in parallel.

**Figure 5:**
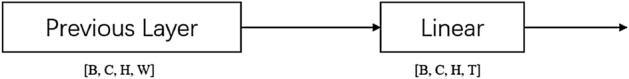
When the length of the target character sequence is greater than the width of the output feature, CNN directly uses full connection to convert the width of the feature from W to T.

In order to solve the above problems, we designed a structure that can flexibly configure the sequence length of the input data of the RNN layer for different target data. The flexible connection layer between CNN and RNN layer, we call it CRFC layer, and its structure is dynamically adjusted based on the characteristics of the output of the CNN layer. For the case where the length of the target character sequence is much smaller than the width of the output feature, its structure is shown in the Fig. 3. The specific process is as follows: (a) the features output by the CNN layer are fed into pooling and fully connected operations, the width W of the feature is transformed into T, and the two are added. We use K to denote the core size of the pooling layer, S to denote the step size of the pooling layer, P to denote the padding of the pooling layer, T to denote the number of sequences we need to input to the RNN layer, and W to denote the width of the feature output by the CNN layer, the calculation formula of K, S, P is as follows:

13 $$\begin{aligned} \left\{ \begin{array}{lr} K = (1, CEIL(W/T))\\ S = (1, CEIL(W/T))\\ T_1 = (CEIL(W/K[1]))\\ P = (0, FLOOR((K[1]*T_1 - W +1)/2)) \end{array} \right. \end{aligned}$$

where $T_1$ represents the number of final sequences of data that we finally get and need to input into the RNN layer. The structure of this layer is shown in Fig. 4, where B represents batch size, C is number of channel, H and W are height and width of feature respectively, and T represents number of sequence. When the length of the target character sequence is greater than the width of the output feature, CNN directly uses full connection to convert the width of the feature from W to T, the structure is shown in Fig. 5.

### Modifications of the convolution layer

In order to improve the recognition effect of narrow characters, the pooling layer of the original CRNN uses a narrow pooling core, but this pooling core does not take into account the wide characters. In the actual data, narrow characters and wide characters coexist. In addition, in order to make the network have stronger feature extraction ability, we improve the CNN layer. The convolutional layer of CRNN-RES is used to extract the feature information of the image, which can be regarded as the feature extraction layer of the network. The purpose of our modification to the convolutional network is to make the network have stronger feature extraction capabilities and ensure that each convolutional layer can extract richer image feature information. As you can see from Fig. 4, compared to the convolutional network of CRNN, we added a layer of BatchNomalization^13^ after the third layer of convolution, in order to make the network better to fit the features of the image, reduce the probability that the features are too complex and exceed the fitting ability of the network. After the convolution of the first layer and the second layer, a pooling layer with a kernel size of $1\times 2$ was added respectively, and the output results of the pooling layer with a kernel size of $2\times 2$ were fused with those of the pooling layer with a kernel size of $1\times 2$. The purpose is to allow the network to use multiple receptive fields to extract features, enhance the feature extraction capabilities of the network, and make the network have better feature extraction capabilities for both wide characters and narrow characters. In the last two pooling layers, we added the pooling layer with the kernel size of $3\times 2$ respectively and fused the output results of the pooling layer with the kernel size of $1\times 2$ and the output results of the pooling layer with the kernel size of $3\times 2$. The purpose here is to make the network use multiple receptive fields to extract features so that the network is friendly to both wide characters and narrow characters. The purpose of adding pooled layers is to obtain different receptive fields and more receptive field characteristics. In this way, the network can better extract the features of narrow characters and wide characters, so as to improve the recognition accuracy of characters of different sizes.

In order to facilitate the reader’s understanding, we will use algebra and graphs to illustrate our changes to the convolutional layer: Assume that the result of maximum pooled branch 1 is A and the result of maximum pooled branch 2 is M. Then the final result P will be obtained by the pooling layer is:

14 $$\begin{aligned} P = A + M \end{aligned}$$

**Figure 6:**
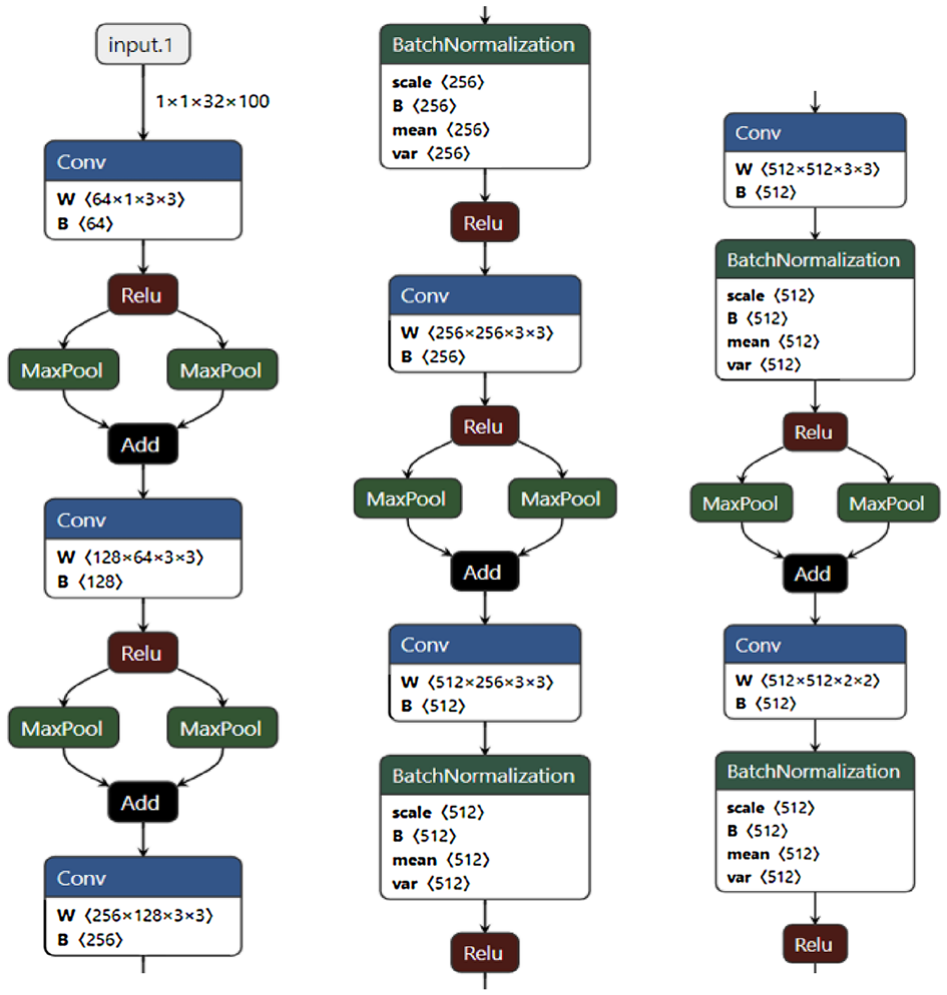
The CNN layer architecture of CRNN-RES.

Our modifications to the CNN module are shown in the following Table 1. Readers can refer to the following Table 1 to understand the detailed structure and parameters of the CNN-RES network. Through the following Table 1, readers can easily understand in detail how we add the Batch Normalization (BN)^13^ layer and the reason of modifying the pooling layer. There are no changes to the CNN module except for adding a Batch Normalization layer and pooling layers. The detailed network structure of the CNN module is shown in Table 1 and the CNN layer architecture of CRNN-RES show in Fig. 6.

**Table 1:** Network structure of the CNN settings.

Network dimension	Model size
Type	Configurations
Transcription	–
Rnn-res	Hidden units: 512
CRFC	
BatchNormalization	–
Convolution	Out-channels: 512, kernel-size: 2, stride: 1, padding: 0
MaxPooling	Kernel-size: $1\times 2$, stride: $1\times 2$/kernel-size: $3\times 2$, stride: $1\times 2$, padding:(1,0)
BatchNormalization	–
Convolution	Out-channels: 512, kernel-size: 3, stride: 1, padding: 1
BatchNormalization	–
Convolution	Out-channels: 512, kernel-size: 3, stride: 1, padding: 1
MaxPooling	Kernel-size: $1\times 2$, stride: $1\times 2$/kernel-size: $3\times 2$, stride: $1\times 2$, padding:(1,0)
Convolution	Out-channels: 256, kernel-size: 3, stride: 1, padding: 1
BatchNormalization	–
Convolution	Out-channels: 256, kernel-size: 3, stride: 1, padding: 1
MaxPooling	Kernel-size: 2, stride: 2/kernel-size: $1\times 2$, stride: $1\times 2$
Convolution	Out-channels: 256, kernel-size: 3, stride: 1, padding: 1
MaxPooling	Kernel-size: 2, stride: 2/kernel-size: $1\times 2$, stride: 2
Convolution	Out-channels: 64, kernel-size: 3, stride: 1, padding: 1
Input	$\hbox {W}\times 32$ gray images

## Evaluation

In this section, we introduce the implementary details, evaluation metrics, and the evaluation results of our method, which includes the comparison results with baseline methods.

### Dataset and hyperparameters

In order to compare with CRNN, and more intuitively show the performance improvement brought by improved network, we chose the same synthetic dataset (Synth)^14^ as the training data. The dataset contains 8 million training images and their corresponding actual words. The same dataset as CRNN was selected for testing, that is ICDAR 2003 (IC03)^4^, ICDAR 2013 (IC13)^5^, IIIT 5k-word (IIIT5k)^6^, and Street View Text (SVT), the test set of these four kinds of data, and the partition of the dataset was not modified. The data used is exactly the same as CRNN. Before the images been input into the network, we also scaled the image uniformly and equally to the size of 100x32. We tested the effects of modifying CNN layer, RNN layer and CRFC layer separately on IIIT5k dataset. Since the maximum character length in IIIT5k is 22, we set the hyperparameters t to 22. All experimental results were calculated using the Tesla V100 GPU.

### Experimental results and discussion

As can be seen from the Tables 2 and 3, the method proposed in this paper is 1.40%, 3.15%, 5.43%, and 2.16% higher than CRNN in the four datasets, respectively. Compared with CRNN, the method proposed in this paper has significantly improved the accuracy. The network model proposed in this paper is 4M smaller than CRNN. The average recognition speed was 1.6% faster than that of CRNN.

**Table 2:** Comparison of recognition accuracy on different data.

Network dataset	IC03	IC13	IIIT5K	SVT
CRNN	0.894	0.867	0.782	0.808
CRNN-RES (ours)	0.969	0.8985	0.8363	0.8296

**Table 3:** Comparison of model size, number of parameters, and recognition speed settings.

Network dimension	Recognition speed (ms)	Number of parameters
CRNN	6.93	8331301
CRNN-RES (ours)	6.80	8197549

We performed ablation experiments to verify the effects of different structures on the model. As shown in Table 4, when CRFC layer is added, the accuracy is improved from 0.782 to 0.813, which proves the effectiveness of CRFC layer. After we use double pooling to modify the CNN layer in CRNN, the accuracy is improved from 0.782 to 0.790, which proves the effectiveness of double pooling to modify the CNN layer. Moreover, after modifying the double-layer BiLSTM of RNN to the single-layer BiLSTM of recursive training, the accuracy is improved from 0.790 to 0.804, and the recognition speed is reduced from 7.01 to 6.71 ms. It is proved that our model can not only improve the accuracy, but also improve the speed of the model.

**Table 4:** Ablation experiments: influence of different network structures on network performance.

Network	CRNN	CRNN + CRFC	CNN + two layer BiLSTM	CNN + one layer LSTM	CNN + one layer RES-LSTM + CRFC
Accuracy	0.782	0.813	0.79	0.804	0.836
Recognition speed (ms)	6.93	7.13	7.01	6.71	6.8

In addition, we show the recognition effect of specific samples in Table 5 and show that our method is more robust in character dense image and wide character image recognition. In short, the CRNN-RES network proposed in this paper achieves a higher recognition accuracy than CRNN under the condition of faster speed and smaller models than CRNN.

**Table 5:**
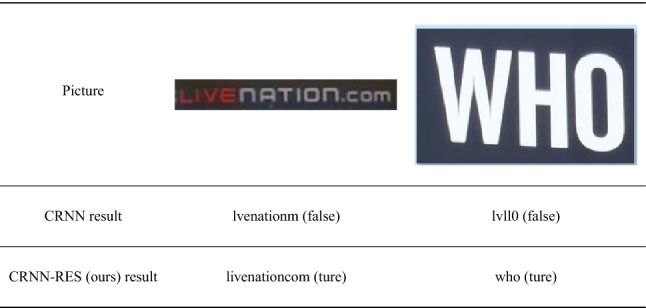
Recognition effect: our method is more robust in character dense image and wide character image recognition.

## Conclusion

This paper introduces a novel neural network method based on BiLSTM to improve the performance of CRNN network. Our method reduce the number of network parameters, while archiving higher accuracy. Extensive experiments on datasets demonstrate the effectiveness of our proposed method.
